# Development and characterization of a multidistance and multiwavelength diffuse correlation spectroscopy system

**DOI:** 10.1117/1.NPh.5.1.011015

**Published:** 2017-09-21

**Authors:** Davide Tamborini, Parisa Farzam, Bernhard Zimmermann, Kuan-Cheng Wu, David A. Boas, Maria Angela Franceschini

**Affiliations:** a Harvard Medical School, MGH/HST Athinoula A. Martinos Center for Biomedical Imaging, Massachusetts General Hospital, Charlestown, Massachusetts, United States; b Boston University, Boston University Neurophotonics Center, Boston, Massachusetts, United States

**Keywords:** near-infrared spectroscopy, diffuse correlation spectroscopy, blood flow, cerebral hemodynamic

## Abstract

This paper presents a multidistance and multiwavelength diffuse correlation spectroscopy (DCS) approach and its implementation to simultaneously measure the optical proprieties of deep tissue as well as the blood flow. The system consists of three long coherence length lasers at different wavelengths in the near-infrared, eight single-photon detectors, and a correlator board. With this approach, we collect both light intensity and DCS data at multiple distances and multiple wavelengths, which provide unique information to fit for all the parameters of interest: scattering, blood flow, and hemoglobin concentration. We present the characterization of the system and its validation with phantom measurements.

## 1 Introduction

Diffuse correlation spectroscopy (DCS) is a relatively new near-infrared spectroscopy (NIRS) method that is being increasingly adopted because of its ability to directly measure an index of microvascular blood flow (${\mathrm{BF}}_{i}$). In particular, its utility to measure cerebral blood flow is now being tested in several clinical applications.^1^^–^^3^ By employing a long coherence length laser and photon counting detectors, DCS measures how fast coherent light loses coherence because of the movement of red blood cells. The correlation diffusion equation ^2^^,^^4^^,^^5^ relates the motion of red blood cells in vessels to the measured temporal autocorrelation decay. The correlation diffusion equation in addition to blood flow also depends on tissue absorption ($\mu_{a}$) and reduced scattering ($\mu_{s}^{\prime }$) coefficients. Hence, by fitting the equation to the measured autocorrelation function, we can derive a quantitative blood flow index (${\mathrm{BF}}_{i}$, ${\mathrm{cm}}^{2}/s$)^6^ only if we know or assume absorption and scattering. It has been demonstrated that the cross talk between static (absorption and scattering) and dynamic (flow) properties of the tissue does not permit fitting for multiple parameters using a single autocorrelation function.^7^ To correctly estimate absolute ${\mathrm{BF}}_{i}$, it is customary to use NIRS in conjunction with DCS^8^^,^^9^ and to simultaneously quantify tissue optical properties and ${\mathrm{BF}}_{i}$. The need for combining two modalities makes the approach more complex.

A recent study^10^ demonstrates how both optical properties and ${\mathrm{BF}}_{i}$ can be estimated with DCS alone by taking advantage of a multidistance approach. It requires the acquisition of autocorrelation curves at different source–detector separations (ρ) and fitting over time delay and ρ. This method can simultaneously fit for $\mu_{a}$, $\mu_{s}^{\prime }$, and ${\mathrm{BF}}_{i}$, but provides robust estimates only in the case of short separations (i.e., ρ<10  mm). While this can work in rodents and small animals, it is not suitable for human measurements where separations larger than 10 mm are required to increase sensitivity to deeper tissues, including cerebral blood flow through the intact skull.

To overcome this limitation, we conceived an approach that, in addition to multiple distances, employs DCS at multiple wavelengths to recover both optical properties and ${\mathrm{BF}}_{i}$ using large separations (20 to 30 mm). The measurement of the intensity decay over distance provides a slope proportional to the product $\mu_{a}\cdot \mu_{s}^{\prime }$ (the effective attenuation coefficient). The measurements of the decay of the autocorrelation function at three or more wavelengths provide the remaining information to uniquely determine reduced scattering, blood flow, and hemoglobin concentration.

In this paper, we present a DCS system able to simultaneously measure optical properties and ${\mathrm{BF}}_{i}$ taking advantage of the multidistance and multiwavelength (MD-MW) DCS approach. We have validated the method’s robustness with tissue-like phantom experiments.

## 2 Theory and Methods

### 2.1 Multidistance Multiwavelength Diffuse Correlation Spectroscopy Method Theory

DCS measures the temporal speckle fluctuations due to the moving scatterers in tissue (red blood cells), which in turn could be used to estimate an index of blood flow in the microvasculature.^4^^,^^6^^,^^11^ The dynamic motion of the medium can be determined by measurement of the autocorrelation function, as faster motion of the scatterers is indicated by faster speckle fluctuations (i.e., more rapid decay of the autocorrelation function). The Green’s function solution of the correlation diffusion equation for semi-infinite boundary conditions^8^ is

$$G_{1}(\rho ,\tau ,\lambda )=\frac{3\mu_{s}^{\prime }(\lambda )}{4\pi }[\frac{e^{-K(\tau ,\lambda )r_{1}(\rho ,\lambda )}}{r_{1}(\rho ,\lambda )}-\frac{e^{-K(\tau ,\lambda )r_{b}(\rho ,\lambda )}}{r_{b}(\rho ,\lambda )}],$$ (1)

where

$$r_{1}(\rho ,\lambda )=\sqrt{1/\mu_{s}^{\prime }{\left(\lambda \right)}^{2}+\rho^{2}},$$ (2)

$$r_{b}(\rho ,\lambda )=\sqrt{{\left(2z_{b}+\frac{1}{\mu_{s}^{\prime }(\lambda )}\right)}^{2}+\rho^{2}},$$ (3)

$$K(\tau ,\lambda )=\sqrt{3\mu_{a}(\lambda )\mu_{s}^{\prime }(\lambda )+6\mu_{s}^{\prime }{\left(\lambda \right)}^{2}k_{0}^{2}(\lambda ){\mathrm{BF}}_{i}\tau },$$ (4)

$z_{b}=2/\mu_{s}^{\prime }(1+R_{\mathrm{eff}})/(1-R_{\mathrm{eff}})$, $R_{\mathrm{eff}}$ is the effective reflection coefficient to account for the index mismatch between tissue and air, $k_{0}=2\pi /\lambda$ is the wave number of light in the medium, corrected λ is the light wavelength, τ is the delay time, ρ is the source–detector separation, ${\mathrm{BF}}_{i}$ is the quantitative blood flow index, $\mu_{a}$ and $\mu_{s}^{\prime }$ are, respectively, the absorption and reduced scattering coefficients. The blood flow index is historically described as the probability of a dynamic scattering event (i.e., scattering from a red blood cell) times the mean square displacement of the dynamic scatterers (i.e., red blood cells).^2^^,^^4^ We recently showed that it can be explicitly related to the absolute blood flow as given by Eq. (15) in Ref. 11, where the probability of scattering from a red blood cell has a potential wavelength dependence given by the blood reduced scattering coefficient divided by the tissue reduced scattering coefficient. This potential wavelength dependence is negligible as both the blood and tissue scattering coefficients vary with wavelength in a similar way. We therefore ignore this potential wavelength dependence here, but will discuss its potential impact in the discussion section. Then, in our homogeneous dynamic phantom measurements (described below), the probability of a dynamic scattering event is equal to 1 and is not wavelength dependent.

DCS measures the normalized intensity autocorrelation function ($g_{2}$), while the correlation diffusion equation applies to the electric field autocorrelation function. To fit the theory to the experimental data, the normalized intensity autocorrelation function must be related to the normalized electric field temporal autocorrelation ($g_{1}$) through the Siegert relation^12^

$$g_{2}(\tau ,\rho ,\lambda )=1+\beta g_{1}{\left(\tau ,\rho ,\lambda \right)}^{2},$$ (5)

where β is a constant determined primarily by the optics of the experiment and it is related to the number of modes in the detected light. In most DCS experiments, employing coherent, nonpolarized sources, and single mode detector fibers, β is ∼0.5.^13^

We aim to decouple the contribution of static (absorption and scattering) and dynamic (flow) properties of the tissue at large separations, which enables us to simultaneously estimate ${\mathrm{BF}}_{i}$, hemoglobin oxygenation (${\mathrm{SO}}_{2}$), and oxygenated and deoxygenated hemoglobin concentrations (HbO and HbR, respectively). To this end, assuming a homogeneous medium, we use the DCS information (light intensity and $g_{2}$ curves) obtained from multiple wavelengths at multiple source–detector separations to fit for the desired parameters ($\mu_{a}$, $\mu_{s}^{\prime }$, and ${\mathrm{BF}}_{i}$).

By measuring the light intensity at each separation and wavelength, I(ρ,λ), and by calibrating sources and detectors, for each wavelength, we obtain the effective attenuation coefficient

$$\mu_{\mathrm{eff}}(\lambda )=\sqrt{3\mu_{s}^{\prime }(\lambda )\cdot \mu_{a}(\lambda )},$$ (6)

since $\mu_{\mathrm{eff}}(\lambda )$ is given by the slope of the simplified solution of the diffusion equation^8^ versus distance

$$\ln[\rho^{2}I(\rho ,\lambda )]=-\mu_{\mathrm{eff}}(\lambda )\rho +I_{0}(\rho =0,\lambda ).$$ (7)

To aid convergence of the fitting algorithm, we apply additional constrains. First ${\mathrm{BF}}_{i}$ is constant over wavelengths, since flow is a mechanical property of the medium and does not depend on the wavelength used to perform the measurement. Then, we take into account the wavelength dependence of $\mu_{s}^{\prime }$ and $\mu_{a}$. The reduced scattering coefficient in tissue follows an empirical power law relationship:^14^^–^^18^

$$\mu_{s}^{\prime }(\lambda )=a\lambda^{-b},$$ (8)

where a is the scaling factor and b is the scattering power, both independent from λ. The absorption coefficient in tissues linearly depends on the hemoglobin concentrations as

$$\mu_{a}(\lambda )=\varepsilon_{\mathrm{HbO}}(\lambda )\cdot \mathrm{HbO}+\varepsilon_{\mathrm{HbR}}(\lambda )\cdot \mathrm{HbR}+p_{H_{2}O}\cdot \mu_{a(H_{2}O)}(\lambda ),$$ (9)

where ε(λ) is the wavelength-dependent oxy- and deoxy-hemoglobin extinction coefficients, obtained from the literature,^19^ and $p_{H_{2}O}$ is the assumed percent of water in tissue.^20^^,^^21^

The MD-MW DCS global fitting is performed to fit experimental data over λ, τ, and ρ to minimize the cost function ($\chi^{2}$) to fit for ${\mathrm{BF}}_{i}$, a, b, HbO, and HbR, that are independent from wavelength and distance

$$\chi^{2}=\overset{N_{\lambda }}{\underset{k=1}{\sum }}\frac{1}{N_{\tau }}\{\overset{N_{\rho }}{\underset{j=1}{\sum }}|\overset{N_{\tau }}{\underset{m=1}{\sum }}{\left[g_{1}^{\text{theory}}(\rho_{j},\tau_{m},\lambda_{k},{\mathrm{BF}}_{i},a,b,\mathrm{HbO},\mathrm{HbR})-g_{1}^{\text{measured}}(\rho_{j},\tau_{m},\lambda_{k},{\mathrm{BF}}_{i},a,b,\mathrm{HbO},\mathrm{HbR})\right]}^{2}|+\gamma |\mu_{\mathrm{eff}}^{\text{theory}}(\lambda_{k},a,b,\mathrm{HbO},\mathrm{HbR})-\mu_{\mathrm{eff}}^{\text{measured}}(\lambda_{k},a,b,\mathrm{HbO},\mathrm{HbR})|\},$$ (10)

where γ is a scaling factor that changes the weight of $\mu_{\mathrm{eff}}$ in the fitting procedure. When γ=0, the fitting discards the information from intensity and the fitted $\mu_{\mathrm{eff}}$ does not necessarily match the measured $\mu_{\mathrm{eff}}$. By increasing γ toward higher values, we are enforcing the fitted $\mu_{\mathrm{eff}}$ to match the measured one. The optimal γ depends on the relative noise levels in the intensity and autocorrelation function data. For our system and experiments, γ between 0.05 and 0.5 provides the best estimates of the optical properties and ${\mathrm{BF}}_{i}$. We used γ=0.3 for the phantom experiment results presented below.

Finally, from the five fitted parameters, we can calculate $\mu_{s}^{\prime }$ and $\mu_{a}$ at each wavelength using Eqs. (8) and (9), as well as the total hemoglobin concentration (HbT=HbO+HbR) and oxygenation (${\mathrm{SO}}_{2}=\mathrm{HbO}/\mathrm{HbT}$).

### 2.2 Methods

We validated the MD-MW DCS approach with measurements in tissue-like phantoms.

We used liquid mixtures of water, Intralipid, and black India ink to increase the absorption or scattering of the solution at regular increments. For the absorption titrations, we mixed 40 ml of 20% Intralipid suspension in 1600 ml of water to achieve a scattering coefficient of $5.5\;{\mathrm{cm}}^{-1}$ at 808 nm. There was initially no absorption (beyond the water itself), and progressive amounts of diluted India ink were added to increase optical absorption to 450% of the initial value. For the scattering titrations, we started by mixing 20% Intralipid with water and India ink to achieve an absorption of about $0.03\;{\mathrm{cm}}^{-1}$ at 808 nm. Additional amounts (8 ml) of concentrated Intralipid were added until scattering increased by 150% of the initial value. The liquid mixture was stirred using a magnetic stirrer after every ink or Intralipid step, and it was allowed to come to rest before taking a measurement, about 2 min (monitored by following the return of the DCS autocorrelation function to a stable value). To simulate blood flow changes, we used the same stirrer and left it on at different levels during the measurements to increase the dynamics of the liquid phantom. For simplicity in this article, we call ${\mathrm{BF}}_{i}$ the mean square displacement of the scattering particles within the solution. For these measurements, we used a silicone oil-based solution, much more viscous than the water, and the Intralipid/India ink solutions were used for the absorption and scattering titrations. This silicon-based solution allowed us to perform measurements at nine stirring levels and increase ${\mathrm{BF}}_{i}$ by 1400% from the initial value.

All measurements were done at a constant temperature of 20°C (changes in temperature affect the Brownian motion of the solution). At each titration step, measurements were done for 20 s per wavelength with the MD-MW DCS system. In addition to the measurements at the three MD-MW DCS wavelengths (767, 808, and 852 nm), we used an additional laser at 785 nm (CL-2000 diode pumped crystal, by CrystaLaser) to check potential improvements using four wavelengths. The recovered optical properties were compared to the optical properties simultaneously measured with a commercial frequency-domain near-infrared spectroscopy (FDNIRS) system (MetaOx, ISS Inc.).^22^ The estimated ${\mathrm{BF}}_{i}$ using the MD-MW DCS method was compared with the ${\mathrm{BF}}_{i}$ calculated using the FDNIRS optical properties at corresponding wavelengths. Interpolations based on the phantom spectral wavelength dependence were used for the optical properties at 850 nm. The FDNIRS multidistance method achieved with a combination of different detectors requires calibration to correct for differences in coupling, gain, and fiber transmission of the detectors and this is done with a solid phantom of known optical properties.^23^ Our DCS MD-MW method requires a similar calibration of the light intensity to estimate $\mu_{\mathrm{eff}}$, but because of speckle noise in a solid phantom, it is preferable to use a liquid phantom as a reference in which the speckle intensity rapidly fluctuates and thus we measure the true average intensity with a short temporal average. In fact, DCS measures a single speckle and must average over longer time than the speckle fluctuation time to estimate the average intensity, while NIRS uses larger detection fibers to average over thousands of speckles and thus measures the average intensity directly. Therefore, DCS intensity was calibrated in the liquid phantom on the first titration, using the optical properties recovered by FDNIRS. We verified the consistency of the data when calibrating on the last titration. The DCS and FDNIRS optical probes were immerged in the solution on opposite sides of the beaker container, far enough to avoid cross talk and allow for simultaneous acquisition. Both probes had the same source–detector separations (15, 20, 25, and 30 mm).

## 3 System Description

**Fig. 1:**
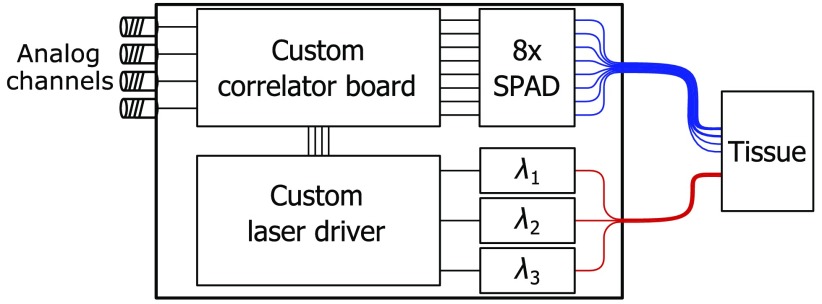
Block diagram of the MD-MW DCS system: three long coherence length lasers at different wavelengths are driven by custom electronics. Eight SPADs collect the light after it propagates through the tissue, and their output signals are sent to a custom-built FPGA-based correlator board. Four analog channels allow for the recording of physiological signals and to evaluate their relationship with the DCS signals.

A block diagram of the MD-MW DCS system is shown in Fig. 1: it makes use of three long coherence lasers at three different wavelengths in the near-infrared spectral range and eight single-photon detectors to collect light at multiple distances. The lasers are driven by custom circuitry and the output light is delivered to the tissue through fiber optics to a source location in the optical probe. The light propagated through the tissue is collected by single-mode fibers located at different distances in the optical probe and delivered to the single-photon detectors. The detector’s outputs are sent to a custom-built field-programmable gate array (FPGA)-based correlator board. Four analog channels are used to record physiological signals. Finally, a USB 3.0 controller is used to transfer DCS and auxiliary data to a remote PC.

### 3.1 Operation Principle

Standard DCS systems use one long coherence length laser operated in CW mode and continuously detect the light at the detector. Multiple detectors are typically used in the same location to average autocorrelation functions and improve signal-to-noise ratio (SNR). In our approach, we need to use three or more lasers at different wavelengths and multiple detectors at different distances. To minimize costs and avoid cross talk between wavelengths, we use a temporal multiplexing approach for turning laser sources on and off in sequence. This approach is intrinsically cross talk free, minimizes the number of detectors needed by employing the same detector for the different colors, and minimizes light losses since it does not require the use of filters to block different wavelengths. The only drawback is the longer measurement time increased by a factor proportional to the number of wavelengths measured. For this approach, we designed and built a laser driver able to provide a stable current to the lasers and to rapidly multiplex the three colors.

We also developed a correlator board that provides the multiplexing signals and performs autocorrelation functions synchronized to each wavelength.

### 3.2 Laser Selection and Driver Circuitry

To implement the temporal multiplexing approach for providing multiwavelengths to the tissue, it is necessary to be able to quickly enable/disable the DCS light sources. We selected the monolithic distributed Bragg reflector (DBR) lasers at 767, 808, and 852 nm (PHxxxDBR series, by Photodigm Inc.). These lasers have a coherence length of tens of meters, a maximum optical power higher than 100 mW when operated in CW, and a subnanosecond turn on/off time that allows also pulsed mode operation.^24^ Each laser is packaged with a thermoelectric cooler (TEC) to keep a stable temperature for improving the laser coherence length.

**Fig. 2:**
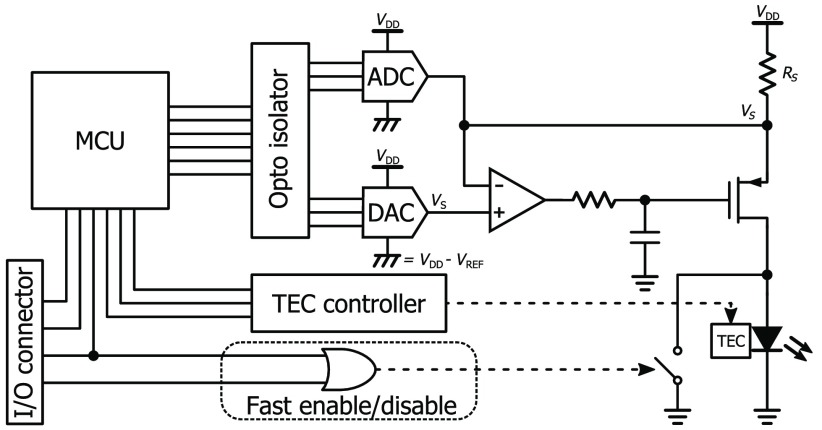
Simplified architecture of laser driver. A current generator is capable of providing to the laser a current between 0 and 500 mA. The generator is isolated and its power supplies are properly filtered for minimizing noise and disturbances. The laser package hosts a TEC to keep a stable temperature for improving the light coherence. A MCU handles the current settings, the temperature of the laser through a TEC controller, and the communication to the system. A fast enable/disable logic allows laser turn on/off for safety reason or for multiplexing the light source.

Figure 2 shows a schematic of the custom-built laser driver based on an ultrastable low-noise current generator capable of providing to the laser a current configurable between 0 and 500 mA. The driver core is basically a standard current generator,^25^ composed of a PMOS transistor able to provide the current set by the sense resistor ($R_{S}$) through a digital set point ($V_{S}$) and an operational amplifier to provide a stable negative feedback. To achieve a long coherence length, we carefully designed the current generator selecting low-noise low-drift components, filtering the supply rails, and minimizing the set point disturbances.^26^

In particular, the 5  Ω sense resistor (Z Series Vishay Foil Resistors, by Vishay) has a 0.05% tolerance and a 0.05  ppm/°C drift, and the operational amplifier (AD8675, by Analog Devices Inc.) has a 0.2  μV/°C drift and only a 2.8  nV/√Hz noise spectral density. The digital set point is provided by a 16-bit digital-to-analog converter (DAC) with a 0.05  ppm/°C drift and an 11.8  nV/√Hz noise spectral density (AD5541A, by Analog Devices Inc.). The DAC is powered between the laser supply ($V_{\mathrm{DD}}$) and $V_{\mathrm{DD}}-V_{\mathrm{REF}}$, where $V_{\mathrm{REF}}$ is a 2.5 V precise voltage reference (VRE3025JS, by Apex Microtechnology Inc.), to minimize the effect of noise and disturbances on $V_{\mathrm{DD}}$. When the set point is configured as $V_{S}=V_{\mathrm{DD}}$, the voltage drop over $R_{S}$ (given by $V_{\mathrm{DD}}-V_{S}$) is 0, resulting in no current flowing into the laser, while setting $V_{S}=V_{\mathrm{DD}}-V_{\mathrm{REF}}$, the voltage drop over $R_{S}$ is $V_{\mathrm{REF}}$, resulting in the maximum current of $V_{\mathrm{REF}}/R_{S}=500\;\mathrm{mA}$ flowing into the laser. The current generator is also isolated and its power supplies are properly filtered to further minimize noise and disturbances.

A microcontroller unit (MCU) (ATMEGA2561, by Atmel Corp.) handles the current settings, the temperature of the laser through a TEC controller (1MD03-024-04/1, by RMT Ltd.), and the communication to the system. There is also a precise 18-bit ADC (analog-to-digital converter) (AD7690, by Analog Devices Inc.) to monitor the current generator and to allow the implementation of a digital control loop. A fast enable/disable logic allows the MCU or an external signal to turn on/off the laser in <100  ns. In this way, the MCU can promptly turn off the laser in case of current generator malfunctioning or the correlator board can provide a signal for fast-multiplexing of the light source.

Finally, simple optics focuses the light into the fiber to connect to the optical probe. An aspheric lens (A375TM-B, by Thorlabs Inc.) collimates the free-space laser’s output and the light passes through an optical isolator (IO-3D-XXX-VLP series, by Thorlabs Inc.) to prevent laser damage due to back reflections. Then, a FiberPort collimator (PAF-X-15-PC-B, by Thorlabs Inc.) focuses the light into the fiber.

### 3.3 Optical Probe

**Fig. 3:**
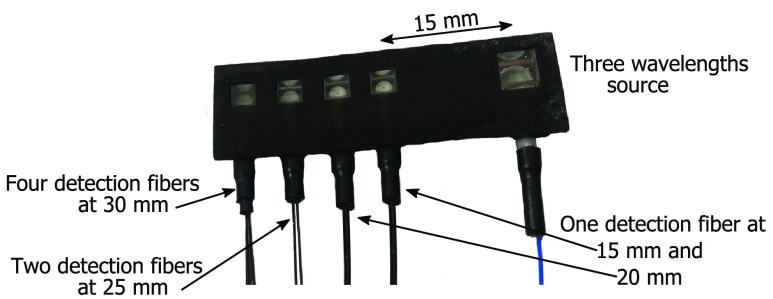
Picture of the DCS probe that includes eight detector fibers distributed in four locations spaced 15 to 30 mm from the source that comprises three source wavelengths. More detection fibers are utilized at longer distances to improve SNR. We offer this and other probe solution to researchers through a nonprofit organization, neuluce.org.

Taking advantage of the single-mode fiber requirement for DCS detectors, we built an ultralight, low-profile, flexible optical probe that easily attaches to the head. To allow for a low probe profile, we use optical prisms in a rubber-like 3-D printed probe head to optimize flexibility and contact with the tissue. Both fibers and prisms are inserted into the probe head, where they are glued with a two-component, medical-grade epoxy featuring very low viscosity and excellent optical-mechanical properties. The first 10 cm of the fibers are protected only by the black plastic coating (125 μm diameter) to maximize flexibility and minimize weight. After that the fibers are combined inside a protective jacket that facilitates handling and safeguards them from possible damage. The resulting probe is shown in Fig. 3. Each laser source is coupled to a 100  μm multimode fiber (numerical aperature=0.39) and attached to the same spot at the optical probe. At the probe end, the light is expanded to a larger area by bonding a 40 deg holographic diffuser between the source fibers and a 5.5 mm prism. The combination of the diffuser and the prism increases the angle of the incident light and minimizes the losses due to backscatter. The light is homogenously spread at the surface of the probe over a 5.5 mm diameter spot. This allows us to use higher optical power (up to 50 mW, resulting in $2.1\;{\mathrm{mW}/\mathrm{mm}}^{2}$) while remaining within the American National Standards Institute (ANSI) maximum permissible exposure of skin to laser radiation: between 2.6 and $4\;{\mathrm{mW}/\mathrm{mm}}^{2}$ in the 760 to 850 nm range, as reported in the ANSI Standard Z136.1-1993 Table. Each detector is coupled to a 4.4  μm single-mode fiber to enable detection of single speckles. The detector fibers at the probe end are glued to 3 mm prisms at different distances from the source. Since the intensity of the detected light exponentially decreases with distance, for longer distances we use multiple detector fibers coupled to the same prism to improve the SNR.^27^

### 3.4 Detectors

The light collected at the probe is sent to single-photon avalanche diode (SPAD) sensors able to detect light with single-photon sensitivity. Fast photon counting is required to measure the autocorrelation function. Key aspects to consider in the selection of these detectors are the photon detection efficiency (PDE) to maximize the detection of the collected light, the dark count rate (DCR) to optimize the SNR, and the afterpulsing probability and the linear relationship between input light and output count rate to minimize distortions when computing the DCS correlation curve.

The detectors employed (SPCM-850-14-FC, by Excelitas Technologies) have a PDE higher than 64% at 767 nm and higher than 54% at 852 nm. These detectors also have a low dead time (20 ns), resulting in an up to 40 Mcps count rate, allowing for a high SNR thanks to a low DCR, which is <100  cps. The afterpulsing probability is <3% and the detectors provide a linear relationship between input light and output count rate for up to 200 kcps, while at 1 Mcps there is a 2% distortion. Further characterization is necessary to determine the maximum conversion rate that guarantees a negligible distortion in the autocorrelation curve.

### 3.5 Correlator Board

**Fig. 4:**
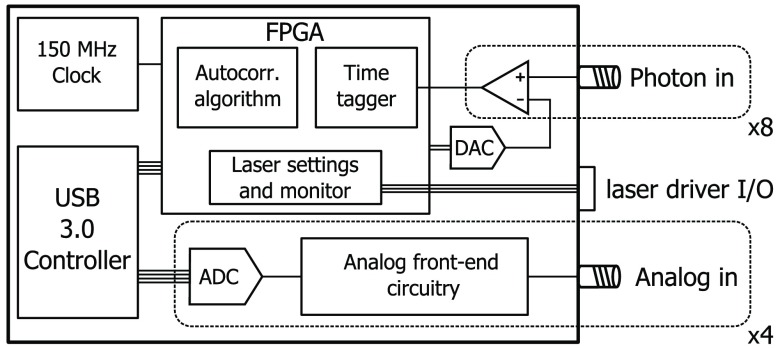
Block diagram of the correlator board. An FPGA time-tags the detected photons using a 150 MHz clock. This allows for performing real-time autocorrelation functions. The board also handles the laser drivers and it acquires four analog channels for recording physiological signals (i.e., blood pressure, ECG, etc.). A USB 3.0 controller transfers all data to a PC.

The last main block of this system is a custom-built correlator board, shown in Fig. 4. The correlator is based on an FPGA device that also hosts eight fast-comparators to translate the single-photon detector outputs to a proper pulse for the FPGA and four analog channels to record analog traces. The FPGA time-tags each detected photon with an arrival time, by means of a counter locked to a 150 MHz clock, used as the time base. For maximum flexibility in the analysis, time gating and autocorrelations are currently not implemented in the FPGA, but are instead performed by software^28^^,^^29^ implementing a multitau scheme, after the data are transferred through a USB 3.0 interface. This allows us to select the integration time in postprocessing, based on the measurement SNR. Because the acquisition rate is not limited by the software, we can quickly calculate autocorrelation functions (at a rate >100  Hz), resulting in blood flow measurements with better than 10 ms resolution. This feature allows us to measure fast blood flow dynamics, which enable better physiological noise filtering and quantification of additional parameters.^30^

The correlator board also handles the light source multiplexing and configures the laser currents to provide the same output power at each wavelength by setting the right driver current, since the output power versus current curve is different for each laser. Finally, by controlling the light multiplexing, the correlator board also tags the photons at different wavelengths to guide the analysis.

## 4 Results

A full characterization of the system was made by testing both the main components and the multidistance multiwavelength method with tissue-like phantoms experiments.

### 4.1 Detection and Correlator Performance

**Fig. 5:**
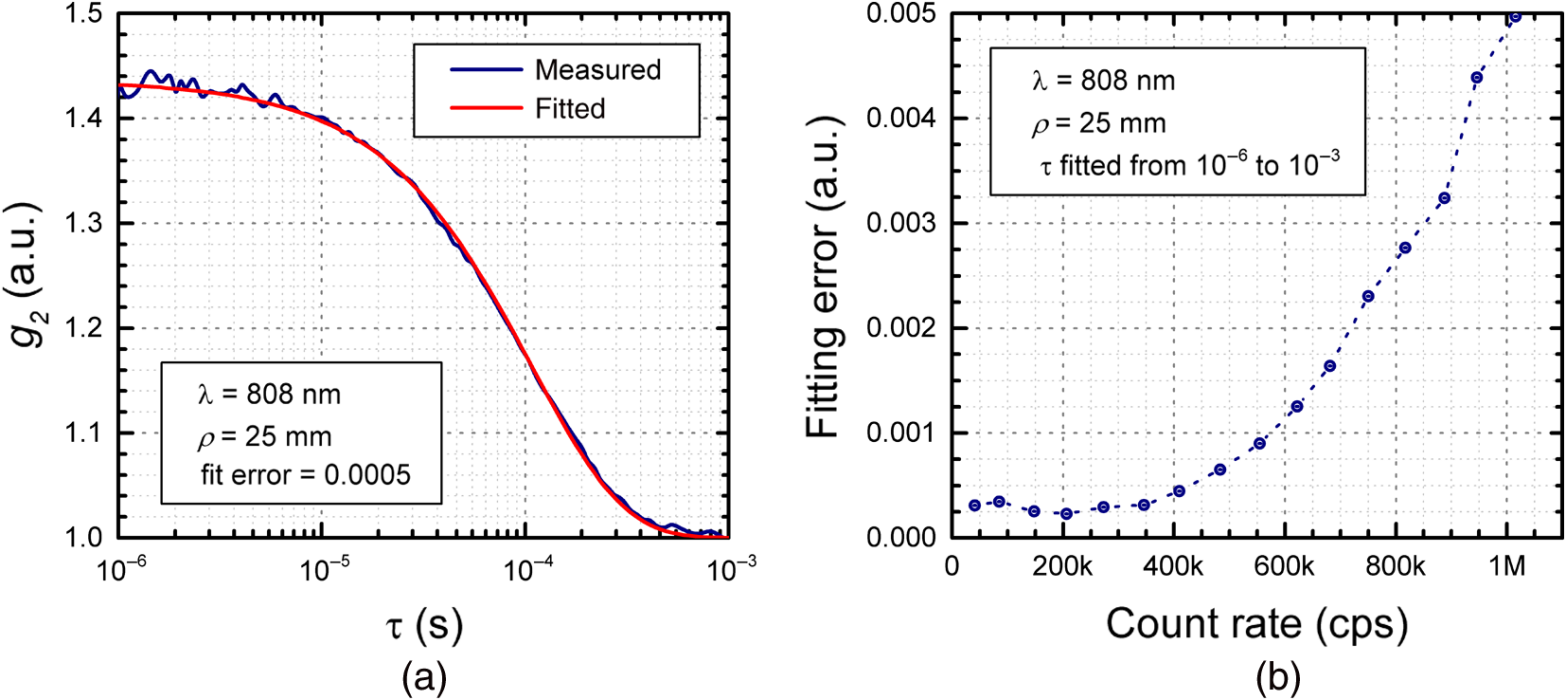
(a) DCS measurement on a microsphere phantom (1 s integration time) and the fitting result. The computed mean squared displacement of $2.58\times 10^{-9}\;{\mathrm{cm}}^{2}/s$ is in agreement with the expected value of $2.5\times 10^{-9}\;{\mathrm{cm}}^{2}/s$, measured using the gold-standard FDNIRS system. (b) Fitting error versus the detector count rate obtained by increasing the light intensity and acquiring DCS autocorrelation curves for 16 different count rate levels between 50 kcps and 1 Mcps. For each step, the autocorrelation functions are computed using the same number of photon (5 million photons).

The detector performance has a significant impact on the quality of the DCS measurements. First, we verified the low detection noise and confirmed a DCR of 80 to 100 cps for all eight detectors. Then, we tested the $g_{2}$ computation by performing measurements with a microsphere liquid phantom using our 808 nm DBR laser. We adjusted the light intensity to get a count rate of 200 kcps to operate the detector in the linear region. The resulting acquired normalized intensity autocorrelation function, $g_{2}$, and its postprocessing fit are shown in Fig. 5(a). We verified that the estimated ${\mathrm{BF}}_{i}$ matched the microsphere proprieties with a low fitting error, computed as the squared norm of the fitting residual divided by the number of τ values. Finally, we characterized the potential impact of the nonlinearity of the detector at high count rates (above 200 kcps) on the autocorrelation function to find the maximum count rate able to guarantee a negligible distortion in the $g_{2}$ curve.

In DCS measurements, the SNR increases with the number of photons used to compute a $g_{2}$ curve. Therefore, by keeping a constant integration time, a higher detection count rate results in higher SNR of the autocorrelation function. The nonlinearity of the detector at high count rates results in a distortion of the $g_{2}$ curve decay. Hence, we increased the light intensity, resulting in count rate between 50 kcps to 1 Mcps and computed the $g_{2}$ curves using the same amount of photons (5 million) to get the same SNR and evaluate only the distortion effect. In Fig. 5(b), we report the fitting error versus the detector count rate. The error, computed using the cost function (simplified version of Eq. (10), with γ=0 and fitting only over τ), is constant for count rates up to 400 kcps and then rapidly increases an order of magnitude at 1 Mcps. To minimize distortion, the resulting optimal device operation is for a count rate no higher than 400 kcps.

**Fig. 6:**
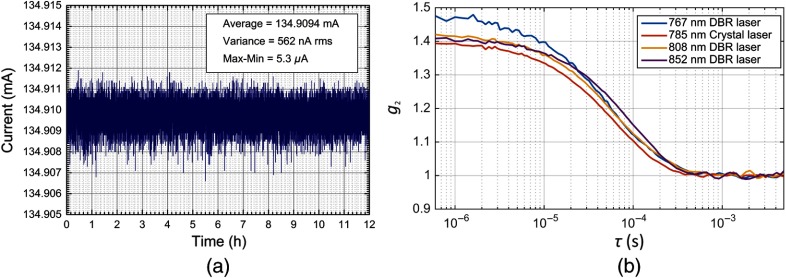
Current stability of the laser driver (a) over 12 h with a temperature fluctuation of about 4°C. Autocorrelation functions (b) of the three DBR laser at 767, 808, and 852 nm, chosen for the system compared to a 785 nm CrystaLaser laser typically used for DCS measurements. The decays are different due to different optical properties of the sample at different wavelengths. The similar β indicates sufficient coherence length of the DBR lasers.

### 4.2 Laser Stability and Coherence

To successfully perform DCS measurements, stable long coherence length sources are required. We first verified the stability of our custom laser driver by measuring the current using a low-drift sense resistor (Z Series Vishay Foil Resistors, by Vishay) as load and then acquiring its potential difference with a 6½ digit resolution digital multimeter (34401A, by Agilent). To achieve the maximum resolution of this multimeter, we acquired the voltage across the resistor with a 2 sps rate for 12 h, at room temperature (between 22°C and 26°C). The current is very stable, as shown in Fig. 6(a), without temperature drifting, thanks to the very low drift electronic components employed. In fact, for current set to 134.9 mA, we measured an average current of 134.9094 mA with a 562 nA root mean square standard deviation, resulting in less than 5 ppm variance.

To test the coherence of the three DBR lasers, we performed a DCS measurement on a liquid phantom, with a fixed source–detector distance of 25 mm, and comparing the three DBR lasers with results from a CrystaLaser laser at 785 nm (CL-2000 diode pumped crystal, by CrystaLaser) typically used for DCS measurements. The autocorrelation functions ($g_{2}$) obtained with the four lasers are shown in Fig. 6(b), resulting in a β close to the theoretical maximum value of 0.5, for all lasers. The differences in decays are due to the different optical proprieties of the liquid phantom at the four wavelengths. This measurement proves that the coherence length of the DBR lasers is sufficient for DCS measurements.

### 4.3 Laser Switching Performance

**Fig. 7:**
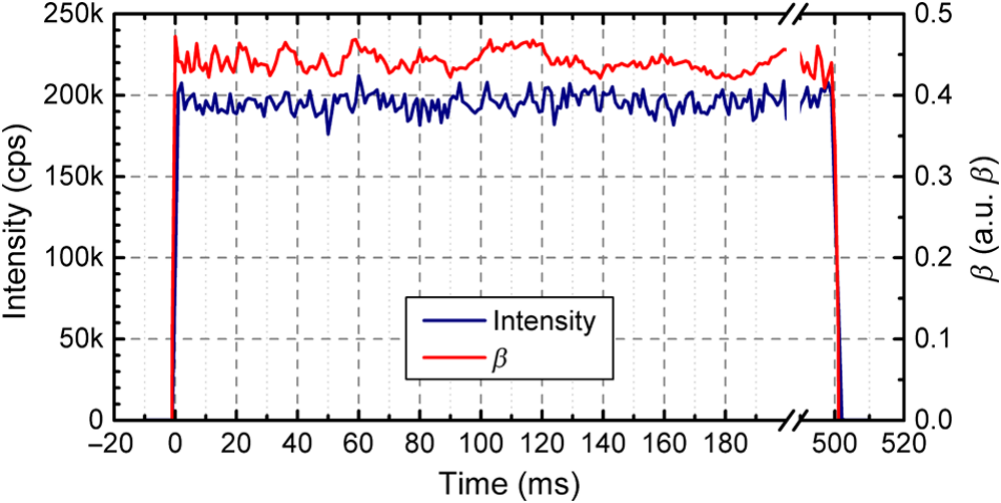
Measurement of the intensity and the coherence factor (β) of the light when switching the 808 nm laser with a 1 Hz square wave. Both intensity and beta are stable within 1 ms of the laser triggering signal.

We measured the behavior of the DBR lasers when turning them on and off in rapid sequence to test whether the coherence length decreases with fast switching. We acquired data on a microsphere phantom and fed a 1 Hz trigger signal with 50% duty cycle to the 808 nm DBR laser to turn it on and off. We acquired the trigger signal through the analog channel to synchronize the $g_{2}$ curves with the laser turn-on times. We computed $g_{2}$ curves and light intensity every 1 ms, from 20 ms before the detected rising edge to 520 ms after it. Since every 1 ms step has a limited number of photons (i.e., 200 photons for 200 kcps) resulting in a very noisy $g_{2}$ curve, we averaged the intensity and the $g_{2}$ curves over 900 periods. The coherence is evaluated as the β of the fitted autocorrelation curves. The behavior of the 808 nm laser is shown in Fig. 7, where both coherence and intensity are stable within 1 ms after the laser turn-on time, with a beta of 0.43±0.02 (mean value±standard deviation computed in the 900 periods) and a count rate of 196±13  kcps. Similar performances were observed with the other two lasers: beta of 0.41±0.02 with a 187±12  kcps count rate at 852 nm and a 0.47±0.03 beta with count rate of 242±17  kcps at 767 nm.

This shows a negligible system warm-up time when the laser is switched on, allowing us to use a multiplexing time as short as tens of milliseconds and to rapidly acquire ${\mathrm{BF}}_{i}$ at the three wavelengths in succession. We also evaluate the impact of the temporal multiplexing approach on the computation of the $g_{2}$ curves. Compared to a standard single-wavelength DCS system, to get ${\mathrm{BF}}_{i}$ with the same acquisition rate with this system, we need to compute three $g_{2}$ curves (one per wavelength) in 1/3 of the time per wavelength. As described in Ref. 31, the noise of the $g_{2}$ curves increase proportionally to $\sqrt{t}$, where t is the integration time. However, we could have excess noise as we also want to rapidly multiplex between wavelengths to make the measurements as nearly simultaneous as possible, but then integrate across multiplexed states to obtain a better SNR. The excess noise would arise from incomplete sampling of the correlation function if we multiplex on time scales approaching the decorrelation time scale. We experimentally verified that switching the lasers on and off at 25 ms intervals do not further increase the noise in $g_{2}$ with respect to CW operation. This was expected as 25 ms is much longer than the typical $10^{-5}$ to $10^{-4}$ decay time for our measured correlation functions.

### 4.4 Phantom Validation

**Fig. 8:**
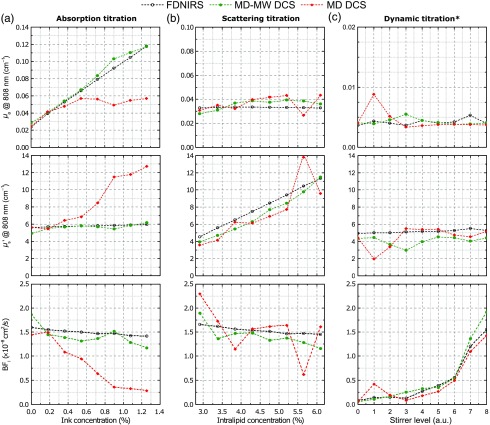
(a) Results on liquid phantoms, of absorption titration, (b) scattering titration, and (c) dynamic titration. Absorption coefficient at 808 nm, scattering coefficient at 808 nm, and blood flow index are recovered using the FDNIRS (black), the multidistance DCS method (red), and the multidistance and multiwavelength DCS (green) methods. For both FDNIRS and DCS, we considered source–detector separations of 15, 20, 25, and 30 mm. *Diffusion titration results are shown at 785 nm due to a failure of the 808 nm and its manufacture long fixing time which has prevented us to use it for these measurements.

To experimentally demonstrate the robustness of the MD-MW DCS method, we performed measurements on liquid phantoms while changing their optical proprieties and simultaneously fitting for a, b, ink concentration, and ${\mathrm{BF}}_{i}$. We compared the results of the MD-MW DCS method with the results obtained using only the multidistance information (MD DCS) and used the absorption and scattering measured with the FDNIRS system and the derived ${\mathrm{BF}}_{i}$ using these coefficients as the reference values.^32^ In Fig. 8 from left to right, we show the absorption, scattering, and dynamic titrations, and from top to bottom, we show the absorption coefficient, the reduced scattering coefficient, and the mean square displacement of the solution as a function of titration level. For the absorption titration, the computed absorption coefficients recovered with our MW-MD DCS approach linearly increase with the Ink concentration and are in good agreement with the FDNIRS values with a maximum deviation of about 25%. The estimated reduced scattering coefficient and ${\mathrm{BF}}_{i}$ remain relatively constant during the absorption titration, revealing small cross talk with changes in absorption. For the scattering titration, the computed reduced scattering coefficients obtained with our method linearly increases with Intralipid concentration, in agreement with the FDNIRS results. The estimated absorption coefficient using the MD-MW DCS method remains relatively constant, while the ${\mathrm{BF}}_{i}$ shows a slight decrease with increased scattering on both FDNIRS and MD-MW DCS. For the dynamic titration, the computed mean square displacement (${\mathrm{BF}}_{i}$) increases with the stirrer level and the recovered absorption and scattering coefficients remain relatively constant during the titration. For all titrations, the parameters derived with the MD-MW DCS method are in good agreement with the parameters computed using the FDNIRS, with a maximum difference of about 25%. The only exception is during stirrer level 3 for which the absorption coefficient differs by about 33%, and the reduced scattering coefficient differs by about 66% between the two modalities. By only using the multidistance DCS information (red traces), we consistently obtained large deviations with the FDNIRS method, with differences in all computed parameters of up to 100% to 400%. These phantom measurements demonstrate that by adding the measures of intensity at multiple distances and DCS at multiple wavelengths, we add unique information that improves the ability to estimate $\mu_{a}$, $\mu_{s}^{\prime }$, and ${\mathrm{BF}}_{i}$, reaching a performance close to the state-of-the-art FDNIRS-DCS method.

## 5 Discussion and Conclusion

We designed and characterized a stand-alone multidistance multiwavelength DCS system able to simultaneously measure tissue optical proprieties and blood flow index, by taking advantage of both the light intensity decay over source–detector separation and the autocorrelation function at three wavelengths.

We demonstrated the capabilities of the MD-MW DCS method by measuring the optical properties and dynamics of liquid phantoms. We compared the results with simultaneous measurements obtained by a commercial FDNIRS system (MetaOx, ISS Inc.).^23^ The absolute values recovered are in agreement with the “gold-standard” FDNIRS-DCS method. The variation of absorption and scattering coefficients with Ink and Intralipid concentration displays excellent linearity. During the scattering titration, the cross talk into absorption is negligible, and the slight decrease in ${\mathrm{BF}}_{i}$ may be due to an increase in viscosity with increasing Intralipid concentration, as ${\mathrm{BF}}_{i}$ also decrease when using the optical properties derived from FDNIRS, to compute ${\mathrm{BF}}_{i}$. Also, when increasing the stirrer level to increase ${\mathrm{BF}}_{i}$ the recovered values are in agreement with the commercial FDNIRS values, with some cross talk with the scattering coefficient at high stirrer levels. The next step will be to validate the method *in vivo*, with both animals and humans.

We have shown that this method is less prone to noise than a previous DCS approach based on multidistance measurements alone.^10^ This is because the additional measures of intensity and $g_{2}$ at different wavelengths provide sufficient information to uniquely recover the unknowns (${\mathrm{BF}}_{i}$, a, b, and chromophore concentrations). Multiple wavelengths have been previously used for DCS measurements^33^ but only to recover hemoglobin oxygenation from the light intensities, not to improve the quantitative estimate of ${\mathrm{BF}}_{i}$ by fitting $g_{2}$ at the multiple wavelengths simultaneously.

The minimum number of source–detector separations to implement the multidistance DCS method could be two, in ideal conditions (e.g., on homogeneous phantoms and in simulations). When considering an *in vivo* measurement, it is not sufficient to rely on only two separations, due to random and systematic errors and the nonhomogenous tissue properties. We have extensive experience with the FDNIRS multidistance method and demonstrated that by considering three or more separations we are able to remove poor-quality data, improving the robustness of *in vivo* measurements.^34^^,^^35^ The same conclusion should apply to DCS or CWNIRS multidistance approaches. For this reason, in this DCS system, we increased the number of source–detector separations to four to verify and determine the linearity of the light intensity over distance. As a drawback, the additional source–detector separations result in a higher number of detectors and an increase in the cost of the system.

A similar argument can be made about the number of required wavelengths. By increasing the number of wavelengths, we provide additional information to help the fitting algorithm to converge to a unique solution, and to minimize the cross talk between ${\mathrm{BF}}_{i}$ and reduced scattering coefficient in the autocorrelation function. Limitation in increasing the number of wavelengths is due to the increasing cost of the system and the low number of coherent lasers available in the near-infrared spectral range. We focused our selection based on the commercially available long coherence laser, the detector efficiency, the hemoglobin spectra, the complexity of the fitting algorithm, and minimizing the number of lasers to reduce the cost. In the liquid phantom experiments, we ran our algorithm using from one to four wavelengths. As shown in Fig. 8, one wavelength is not enough to properly recover the parameter, resulting in errors >100%. Using two wavelengths partially reduce these errors to 75%. As shown in Fig. 8, using three wavelengths we obtained consistent improvements. We also verified negligible improvement by adding a fourth wavelength during the absorption and scattering titrations. For these reasons, we consider three wavelengths to be the optimal compromise between performance and cost with this number of unknowns.

Our algorithm minimizes the cost function by fitting not only the measured autocorrelation function, but also the effective attenuation coefficient. The contribution of this last term is determined by γ [Eq. (10)]. In our analysis, a value of γ between 0.05 and 0.50 results in <10% variance of the results. The results presented in Fig. 8 are obtained using γ=0.30, providing a good contribution of the $\mu_{\mathrm{eff}}$ data to the cost function.

With respect to current combined NIRS and DCS systems, using only DCS there are advantages in reducing cost, size, and complexity of the device. For instance, typical NIRS-DCS systems consist of a DCS component and either an FDNIRS^23^ or a time-resolved spectroscopy (TRS) component.^36^ The NIRS and DCS components do not share either sources or detectors, which leads to a more complex system architecture and increased costs and size of the combined system with respect to a stand-alone system. We have recently proposed a TD-DCS system^24^ that uses a pulsed laser and a photon counting detector to quantify both optical properties and blood flow. While the method is very appealing, and will in the future be the best solution, it currently requires very sophisticated components (custom high-power laser, red-enhanced SPAD detectors, and high-performance time-correlated single-photon counting electronics), which does not necessarily reduce costs. Combining DCS with CWNIRS, while simple and inexpensive, does not help with the recovery of the reduced scattering coefficients. As we showed here in experiments, acquiring autocorrelation functions at only one wavelength is not sufficient to separate scattering from blood flow. Without the knowledge of the scattering, DCS can only quantify relative changes in ${\mathrm{BF}}_{i}$.

Another advantage of stand-alone DCS resides in the ability to use very light, flexible, and small optical probes as the patient interface. In fact, the DCS requirement of a single-mode detector fiber (4.4  μm diameter) to collect light from single speckles, instead of the large NIRS fiber bundles, allows us to develop small probes as shown in Fig. 3. This represents a strong advantage for continuous monitoring, since lighter and more flexible fibers and probes can be more easily attached, reducing motion artifact and providing more comfort for the patient. In fact, the fibers we use have high strength (≥200  kpsi) and a very high bending radius (≥6  mm), which allow us to keep the probe in place without forcing it in position. The thin coating and jacket used on the fibers at the probe interface may be prone to damage if mishandled. Still, we do not see this as a major problem since the optical probe needs to be treated with care in any case, as it contains polished fibers and optics and needs to be attached to the patient.

The proposed MD-MW approach to estimate the optical properties from DCS alone is based on the same assumptions used for the multidistance multiwavelength FDNIRS method,^22^^,^^37^ i.e., the investigated tissue is assumed to be homogeneous. For the FDNIRS, this assumption holds in the case of separations >10  mm and superficial layers thinner than 5 to 6 mm, as demonstrated both experimentally in phantoms,^38^ and with Monte Carlo simulations in segmented MRI children head models.^39^ In fact, the MD-MW FDNIRS method is considered by many the gold standard for cerebral oximetry.^32^ In the future, we expect these depth sensitivity results to be replicated for this DCS method. In fact, the work of Selb et al.^40^ indicates that the depth sensitivity of DCS is great than CW NIRS because the early part of the decay of the autocorrelation function is driven by longer path-length photons than the average path length of CW NIRS photons. We expect this method to work best in infants where the homogeneous medium assumption works well. In adults, even with the greater depth sensitivity of DCS, layered models need to be applied to obtain quantitative values. Also in adults, larger source–detector separations will be necessary to estimate optical properties of the cerebral cortex. In this case, we can adopt different strategies to increase SNR at the larger distances. For instance, we can increase the integration time for the recovery of the scattering coefficient. The scattering coefficient changes slowly over time. It is thus sufficient to measure it every 1 to 2 min, and assume it is constant during these periods while fitting for ${\mathrm{BF}}_{i}$ and hemoglobin concentrations at a much faster rate. We can add additional lasers to increase the number of independent measurements in the MD-MW DCS fitting procedure. We can also add channels at larger separations with larger detector fibers for additional intensity measurements, since sensitivity to deeper layers is lower for intensity than for $g_{2}$.^40^

In conclusion, we have presented a new stand-alone DCS system that employs lasers at multiple wavelengths and multiple detector channels to recover both optical properties and blood flow of biological tissues. We have characterized the system and demonstrated initial feasibility of the method.
